# Construction and Validation of Convenient Clinicopathologic Signatures for Predicting the Prognosis of Stage I-III Gastric Cancer

**DOI:** 10.3389/fonc.2022.848783

**Published:** 2022-03-24

**Authors:** Weiqiang You, Zerong Cai, Nengquan Sheng, Li Yan, Huihui Wan, Yongkun Wang, Jian Ouyang, Lu Xie, Xiaojian Wu, Zhigang Wang

**Affiliations:** ^1^ Department of General Surgery, Shanghai Jiao Tong University Affiliated Sixth People’s Hospital, Shanghai, China; ^2^ Department of Colorectal Surgery, The Sixth Affiliated Hospital, Sun Yat-sen University, Guangdong Institute of Gastroenterology, Guangdong Provincial Key Laboratory of Colorectal and Pelvic Floor Diseases, Guangzhou, China; ^3^ Shanghai-MOST Key Laboratory of Health and Disease Genomics, Institute for Genome and Bioinformatics, Shanghai Institute for Biomedical and Pharmaceutical Technologies, Shanghai, China

**Keywords:** gastric cancer, prognosis, risk score, clinicopathologic signature, stage I-III

## Abstract

**Background:**

Patients with stage I-III gastric cancer (GC) undergoing R0 radical resection display extremely different prognoses. How to discriminate high-risk patients with poor survival conveniently is a clinical conundrum to be solved urgently.

**Methods:**

Patients with stage I-III GC from 2010 to 2016 were included in our study. The associations of clinicopathological features with disease-free survival (DFS) and overall survival (OS) were examined *via* Cox proportional hazard model. Nomograms were developed which systematically integrated prognosis-related features. Kaplan–Meier survival analysis was performed to compare DFS and OS among groups. The results were then externally validated by The Sixth Affiliated Hospital, Sun Yat-sen University.

**Results:**

A total of 585 and 410 patients were included in the discovery cohort and the validation cohort, respectively. T stage, N stage, lymphatic/vascular/nerve infiltration, preoperative CEA, and CA19-9 were independent prognostic factors (P < 0.05). Two prognostic signatures with a concordance index (C-index) of 0.7502 for DFS and 0.7341 for OS were developed based on the nomograms. The 3-year and 5-year calibration curves showed a perfect correlation between predicted and observed outcomes. Patients were divided into three risk groups (low, intermediate, high), and distinct differences were noticed (p < 0.001). Similar results were achieved in the validation cohort. Notably, a free website was constructed based on our signatures to predict the recurrence risk and survival time of patients with stage I-III GC.

**Conclusions:**

The signatures demonstrate the powerful ability to conveniently identify distinct subpopulations, which may provide significant suggestions for individual follow-up and adjuvant therapy.

## Background

Gastric cancer (GC) is a major burden on global health, with the fifth morbidity and fourth mortality, especially in China (1, 2). R0 radical resection is the golden standard therapy for non-metastatic GC. However, even after R0 resection, 70% of patients die from recurrence or metastasis, and the 5-year survival rate is only 40%-58% (3). Recurrence and metastasis after R0 surgery are the significant factors affecting the survival of stage I-III GC patients.

At present, the tumor–node–metastasis (TNM) stage according to the 8th AJCC staging system is recognized as a reliable standard prognosticator for adjuvant therapy and prognosis (4, 5). Generally, a higher stage predicts a worse prognosis. However, due to differences in tumor biology and individuals, the prognosis of GC with the same stage is heterogeneous. It suggests that judging the prognosis only based on the TNM stage is incomplete. Several indicators, such as pathological features, preoperative serum tumor markers, and adjuvant therapy, are also meaningful for the prognosis of GC. For example, in node-negative GC, lymphatic and vascular invasion heralded poor prognosis and short survival (6). CEA and CA19-9 are the most common markers used for the early diagnosis and prognosis of GC. Many studies reported that the perioperative levels of CEA and CA19-9 have crucial significances and can be used to monitor recurrence and predict postoperative survival and surgical outcomes (7–13).

Therefore, it is a complex and multidimensional problem to accurately evaluate the prognosis of patients with stage I-III GC after radical surgery. In this study, we attempted to construct clinicopathologic signatures that integrated these prognostic factors to evaluate the prognosis of stage I-III GC accurately.

## Methods

### Data Collection

This study was a double-center retrospective clinical study registered in the Chinese Clinical Trial Registry (Approval No. ChiCTR1900024346). It was approved by the ethics committee of Shanghai Jiao Tong University Affiliated Sixth People’s Hospital (Approval No. 2019-KY-032K). The training cohort from Shanghai Jiao Tong University Affiliated Sixth People’s Hospital was used to construct the signatures, and then the external cohort from the Sixth Affiliated Hospital of Sun Yat-sen University to validate. All the patients underwent R0 radical resection and were pathologically diagnosed with stage I-III GC from January 2009 to December 2016. Written informed consent was waived because of the study design. The exclusion criteria were as follows: (1) stage IV GC; (2) non-adenocarcinoma; (3) no available preoperative serum tumor markers; (4) younger than 18 years old.

Clinical data was mainly provided by medical histories and the electronic medical record department. The pathological stage was accessed by the 8th AJCC criterion for GC. Preoperative serum tumor markers were examined within one week before R0 surgery (including subtotal gastrectomy or total gastrectomy). Gastric tumor sites include cardia, gastric body, and antrum carcinoma. Adjuvant therapy and follow-up were according to National Comprehensive Cancer Network (NCCN) guidelines. Disease-free survival (DFS) was defined as the time from surgery to tumor metastasis or recurrence. Overall survival (OS) was defined as the time from surgery to death.

### Statistical Analysis

Data processing and data analysis were performed in the R language (version 3.4.4). For prognostic signatures, we firstly performed a univariate analysis of all variables by Cox proportional hazards regression. Then, the significant covariates (score test p <0.05) were included in the multivariate Cox proportional hazards regression. Stepwise and backward selection processes were performed to achieve the final signatures according to the Akaike information criterion (AIC). Signature discrimination was examined by the concordance index (C-index) and corrected 1,000 times by bootstrapping. Furthermore, we classified the patients into three groups (high, intermediate, and low risk) based on the risk score. The Kaplan-Meier (K-M) curves were used to compare survival time among groups. The log-rank test was performed to calculate the significant differences. Nomograms were established to predict the survival rates. P values were two-sided, with statistically significant differences at p < 0.05.

## Results

### Patient Characteristics

From January 2009 to December 2016, a total of 995 patients were enrolled from two cohorts according to our inclusion and exclusion criteria. 585 patients with stage I-III GC from Shanghai Jiao Tong University Affiliated Sixth People’s Hospital were screened for the internal cohort to construct the signatures. The external cohort including 410 cases from the Sixth Affiliated Hospital of Sun Yat-sen University was used to validate the signatures (**Supplementary Figure 1**).

Patients’ clinical information and follow-up data from the training cohort were shown in **Supplementary Table 1**. The median age at the time of diagnosis was 63 years. 154 patients were diagnosed with stage I, 165 patients with stage II, and 266 cases with stage III. 557 patients had the pathological type of adenocarcinoma (95.2%), and 28 patients had the worse pathological type. LN metastasis occurred in 324 patients (55.4%). 103 patients had well and moderate tumor differentiation, while the others had poor differentiation. Lymphatic infiltration, vascular infiltration, and nerve infiltration occurred in 386, 141, and 461 patients, respectively. The median Ki67 was 55% (range: 5%-95%). The mean NLR and PLR were 2.92 and 158.12, respectively. The mean levels of CEA, CA19-9, and CA125 were 8.83 ng/ml, 17.47 U/ml, and 6.69 U/ml, respectively. The median follow-up time was 45 months. Recurrence occurred in 203 patients (34.7%). A total of 230 patients (39.3%) died during the follow-up.

### Construction and Internal Validation of Prognosis Signatures

**Figure 1:**
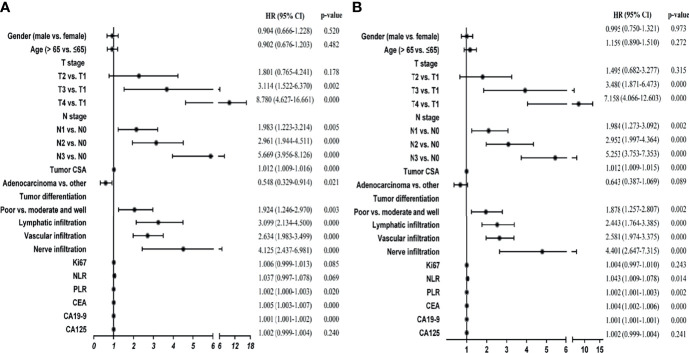
Univariate Cox regression analyses of stage I-III GC patients in the training cohort. **(A)** Univariate Cox regression analyses for DFS. **(B)** Univariate Cox regression analyses for OS.

To select the variables that were suitable for inclusion in our signatures, we performed univariate analyses. Our results suggested that 10 variables, including T stage, N stage, tumor CSA, differentiation, lymphatic infiltration, vascular infiltration, nerve infiltration, PLR, CEA, and CA19-9, were significantly associated with DFS and OS in GC patients (**Figures  1A, B**; p < 0.05). Pathological type affected DFS (**Figures  1A, B**; p < 0.05) but not OS, whereas NLR had the opposite effect. Therefore, there were 11 variables closely related to DFS or OS in GC.

**Figure 2:**
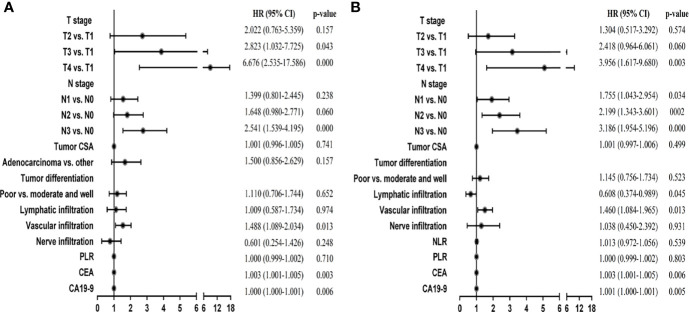
Multivariate Cox regression analyses of stage I-III GC patients in the training cohort. **(A)** Multivariate Cox regression analyses for DFS. **(B)** Multivariate Cox regression analyses for OS.

Then, we used these variables as potential prognostic factors to construct prognostic signatures with the Cox proportional hazards model. AIC, stepwise, and backward analyses were performed as variable selection methods. Finally, 6 indicators were selected for the DFS signature in GC: T stage, N stage, vascular infiltration, nerve infiltration, CEA, and CA19-9. Our multivariate analysis results showed that they were all independent prognostic factors except nerve infiltration (**Figure 2A**; p < 0.05). The discriminative power, shown by the C index (concordance index), was 0.75, corrected with 1,000 permutations, for DFS in GC in the training cohort (**Supplementary Table 2**). Our OS signature in GC also included 6 indicators, namely, T stage, N stage, lymphatic infiltration, vascular infiltration, CEA, and CA19-9. These variables were all independent factors for OS in GC (**Figure 2B**; p < 0.05). The C-index of our OS signature was 0.73, corrected with 1,000 permutations (**Supplementary Table 3**). Overall, we constructed predictive prognostic signatures for DFS and OS in GC.

### Evaluation and Determination of the Accuracy and Predictive Power of the Signatures

**Figure 3:**
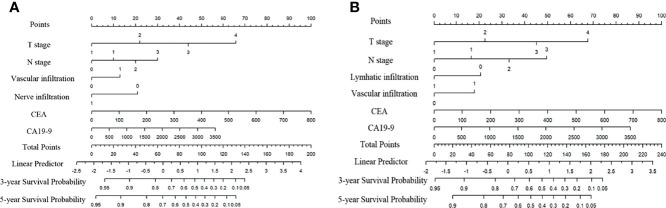
Nomograms for variables from the internal training cohort included in the signatures. **(A)** Nomogram for variables included in the model for DFS in GC. **(B)** Nomogram for variables included in the model for OS in GC. Lymphatic infiltration, vascular infiltration, nerve infiltration: 0-absent, 1-present. Differentiation: 1-high, 2-moderate, 3-low, 4-undifferentiation.

**Figure 4:**
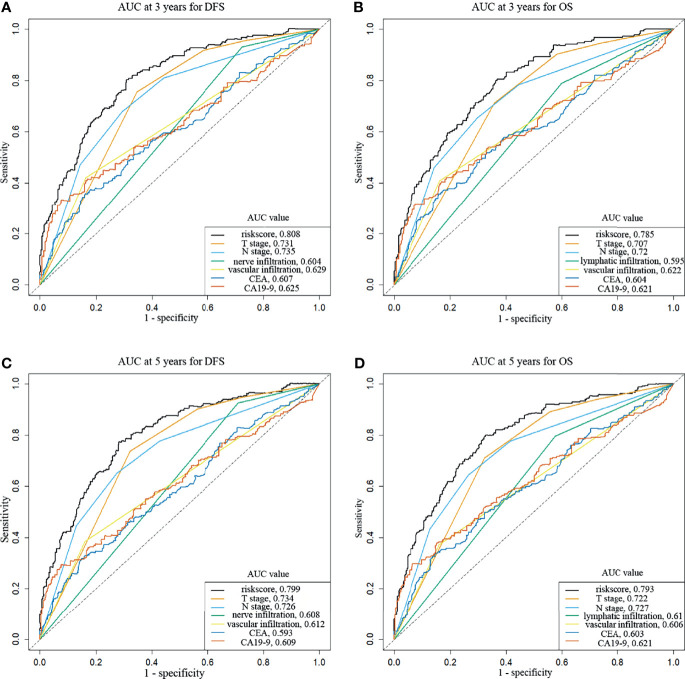
Time-dependent receiver operating characteristic curves show the predictive accuracy of the signatures. **(A)** AUC curves at 3 years for DFS. **(B)** AUC curves at 3 years for OS. **(C)** AUC curves at 5 years for DFS. **(D)** AUC curves at 5 years for OS.

**Figure 5:**
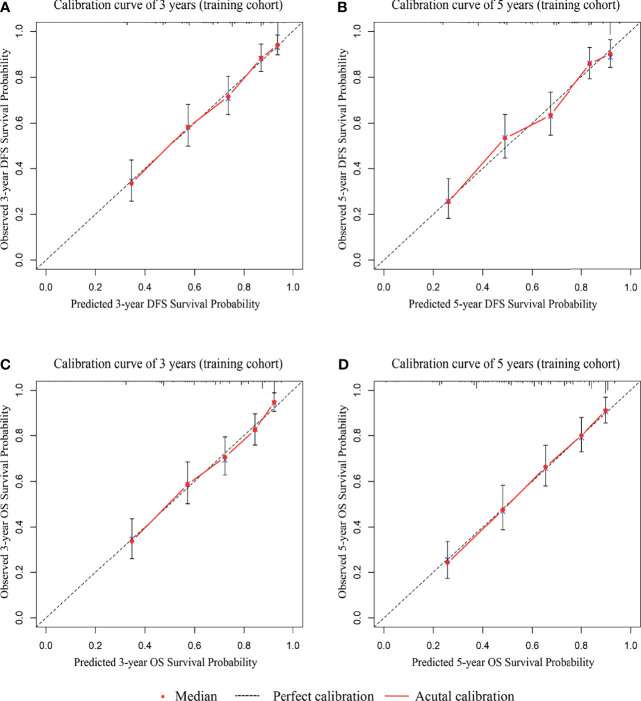
The 3-year and 5-year calibration curves are based on the internal cohort nomograms between predicted and observed DFS and OS outcomes. **(A)** The 3-year calibration curve for DFS. **(B)** The 5-year calibration curve for DFS. **(C)** The 3-year calibration curve for OS. **(D)** The 5-year calibration curve for OS.

To evaluate the prognosis of patients more intuitively, we developed nomograms with Cox regression models. All the variables in the nomogram had a weighted score, and we could predict the 3-year or 5-year survival outcome by the sum of the scores (**Figure 3**). To further verify the importance of these variables and calculate the risk score, we developed a nonparametric approach in our signature using random survival forest. Our results showed that the T stage had the largest influence on DFS and OS with a VIMP of positive value 0.6983 and 0.518, followed by vascular infiltration, N stage, CEA, and CA19-9 (**Supplementary Figure 2**). The predictive accuracy of the signatures using time-dependent ROC analysis was relatively high. The AUC of the DFS signature based on the risk score was 0.808 at 3 years and was 0.799 at 5 years (**Figures 4A, C**), higher than the other single indicator. Similarly, the AUC of the OS signature was 0.785 at 3 years and was 0.793 at 5 years (**Figures 4B, D**). The calibration curves for GC based on our signatures showed an excellent correlation between predicted and observed outcomes for DFS and OS prediction at 3 years and 5 years (**Figure 5**). All these results illustrated that the DFS and OS signatures had good accuracy and prediction ability.

### Prognosis Among Risk Groups With Different Scores

**Figure 6:**
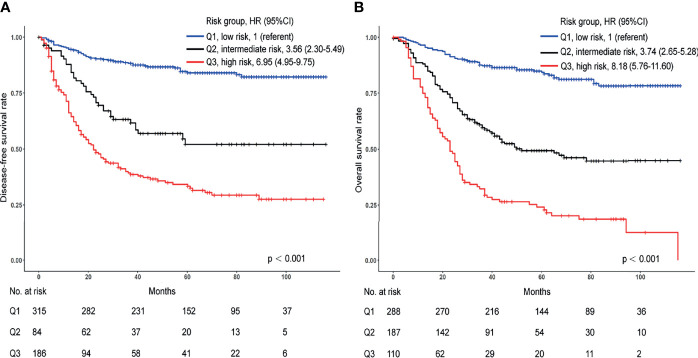
Kaplan-Meier curves for differences in patient survival in the high-, intermediate-, and low-risk groups from the internal cohort. **(A)** Kaplan-Meier curves for DFS. **(B)** Kaplan-Meier curves for OS.

We assessed patients according to the risk score achieved from the signatures using these different variables. Kaplan-Meier curves were applied to compare survival differences. In the DFS signature, compared with the low-risk group, the medium-risk group (HR 3.56; 95% CI, 2.30-5.49) and the high-risk group (HR 6.95; 95% CI, 4.95-9.75) had shorter survival times (**Figure 6A**, p < 0.001). We found similar results for OS signature. Compared with the low-risk group, the intermediate- and high-risk groups had hazard ratios of 3.74 (95% CI, 2.65-5.28) and 8.18 (95% CI, 5.76-11.60), respectively (**Figure 6B**, p < 0.001). Furthermore, the 5-year rate of the low-risk group was 84.0% for DFS and 83.0% for OS, much higher than the intermediate group (53.0% for DFS and 50.0% for OS) and high-risk group (35.0% for DFS and 24.0% for OS).

### Validation of the Signatures in an External Cohort

**Figure 7:**
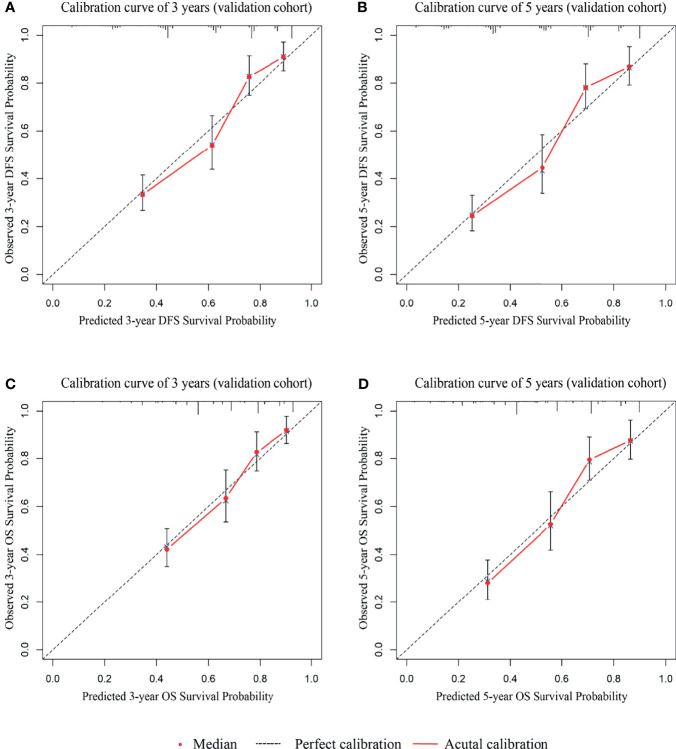
The calibration curves for external cohort validation between predicted and observed DFS and OS outcomes. **(A)** The 3-year calibration curve for DFS. **(B)** The 5-year calibration curve for DFS. **(C)** The 3-year calibration curve for OS. **(D)** The 5-year calibration curve for OS.

To examine whether the signatures were suitable for other centers, we collected data from an external cohort for validation. 410 patients with stage I-III GC were collected. Univariate analysis results showed that the variables included in the predictive DFS and OS signatures were all prognostic factors (**Supplementary Table 4**, p < 0.05) except CA19-9. The calibration curves suggested a perfect correlation between predicted and observed outcomes for DFS and OS prediction at 3 years and 5 years (**Figures 7A–D**). In addition, according to the scoring criteria, we divided the patients from the external cohort into three groups (high, intermediate, and low risk), and similar results were obtained. The DFS and OS curves in the validation cohort among the three groups were significantly different (**Supplementary Figures 3A, B**, p < 0.001).

### Website for Predicting the Prognosis of Stage I-III GC

Based on the signatures, we developed a free website for predicting the prognosis of stage I-III GC (http://115.28.66.83/liuyuan/stad.php).

## Discussion

The postoperative surveillance after R0 resection of stage I-III GC remains pessimistic. Relapse and metastasis always lead to a poor prognosis. At present, postoperative adjuvant treatment and patients’ follow-up are based on the TNM staging system (14, 15). However, to determine the prognosis of GC only by TNM staging system is partial because of the heterogeneity of the tumors at different stages. Many other indicators are also extremely meaningful. Preoperative CEA and CA19-9 have been reported to correlate with the prognosis, and elevated tumor markers indicate metastasis and short survival (8, 10, 12, 13, 16, 17). Besides, the combination of multiple serum tumor markers can improve the accuracy of the prediction compared with a single marker (18). Considering this, we designed this study to construct Nomogram scales and prognostic signatures incorporating these prognostic factors, which could evaluate the risk of postoperative relapse and overall survival effectively. In our study, 995 patients with stage I-III GC from two cohorts were enrolled. Then, we systematically analyzed the significance of different variables on the prognosis and constructed clinicopathologic signatures that predicted postoperative DFS and OS. Our results indicated that the prediction signatures had promising accuracy and predictive ability. Besides, our validation cohort demonstrated that the signatures were widely applicable in clinical.

Currently, many studies have attempted to construct models to accurately evaluate the prognosis of GC. A risk classification model based on the immunohistochemical expression of three proteins (APC, FHIT, and HER2) and five pathological parameters (tumor stage, resected nodes, margins, location, and sex) accurately separates the patients with GC into three groups (19). Using clinically practical information, Jun Lu et al. developed a model incorporating T stage, number of metastatic LNs, M stage, and operative time for accurately determining the 5-year overall survival of remnant GC (20). Sufficient evidence has shown that systemic inflammatory response (SIR) was significant in the occurrence and development of multiple tumors (21, 22). Several studies have found that various inflammatory cells and immune system signaling molecules, such as NLR, PLR, and LMR, is related to GC progression (23, 24). In our study, although univariate analysis found that NLR and PLR affected the prognosis of stage I-III GC, multivariate analysis revealed that they were not independent prognostic indicators. Thus, NLR and PLR were not included in our signatures, which may be due to their insufficient importance. Abnormal DNA methylation plays an important role in the early development of tumors (25). A related study revealed that four DNA methylation signatures could serve as an important tool to predict the prognosis of GC patients (26). Stenholm et al. genotyped seven microRNA polymorphisms and found that these selected miRNAs had a significant impact on the prognosis of advanced GC (27). Tumor immune microenvironment is one of the research hotspots in recent years (28, 29). The concept of Immunoscore provides a new direction and a strong guarantee for the evaluation of various tumors (30–33). Increasing studies have shown that the density of CD3^+^ and CD8^+^ lymphocyte populations in the tumor microenvironment could effectively predict the recurrence and metastasis of GC and complement the TNM staging system (34, 35).

From this point of view, these signatures respectively used different indicators to predict the prognosis of GC. If clinicians want to use these signatures, relevant variables must be examined, which is not only technically demanding but also financially burdensome. Our signatures are convenient without additional testing. These characteristics determine that our signatures have a better application prospect in clinical practice. Patients with stage I-III GC could be evaluated by our signatures before they received postoperative adjuvant treatment and follow-up. Our signatures could help the clinicians to identify patients with high-risk signatures and adequate adjuvant therapy and intensive follow-up recommendations are necessary. However, our research also has several shortcomings. Firstly, it is a retrospective study, and our signatures may need to be verified by prospective studies. Secondly, our signatures do not include the latest markers, such as gene status and Immunoscore. In addition, perioperative adjuvant therapy were not included in our signatures, because not all patients in this study received adjuvant therapy. At last, the sample size included in this study is limited, so our results need to be verified in a larger population. Further studies will be carried out to include biomarkers reflecting the immune microenvironment and indicators such as Immunoscore and microsatellite status to optimize our signatures.

## Conclusions

The signatures including T stage, N stage, lymphatic/vascular/nerve infiltration, CEA and CA19-9 based on nomogram scales could distinguish the prognosis of stage I-III GC patients with different risk scores accurately and have broad clinical application prospects.

## Data Availability Statement

The raw data supporting the conclusions of this article will be made available by the authors, without undue reservation.

## Ethics Statement

The studies involving human participants were reviewed and approved by The ethics committee of Shanghai Jiao Tong University Affiliated Sixth People’s Hospital. Written informed consent for participation was not required for this study in accordance with the national legislation and the institutional requirements.

## Author Contributions

ZW conceived the project. WY, LY, and NS collected the clinical and follow-up data from Shanghai Jiao Tong University Affiliated Sixth People’s Hospital. XW and ZC collected the clinical and follow-up data from the Sixth Affiliated Hospital of Sun Yat-sen University. LX, HW, JO, and YW analyzed all data using the R language. WY wrote the manuscript. All authors contributed to the article and approved the submitted version.

## Funding

This work was supported by National Key Clinical Discipline, Shanghai Municipal Education Commission-Gaofeng Clinical Medicine Grant Support (no.20172023), Shanghai Science and Technology Commission Medical Project (no.16411953200), Shanghai Pujiang Program (no.16PJ1408200), Natural Science Foundation of Shanghai (no.16ZR1449600), National Natural Science Foundation of China (no.81602689).

## Conflict of Interest

The authors declare that the research was conducted in the absence of any commercial or financial relationships that could be construed as a potential conflict of interest.

## Publisher’s Note

All claims expressed in this article are solely those of the authors and do not necessarily represent those of their affiliated organizations, or those of the publisher, the editors and the reviewers. Any product that may be evaluated in this article, or claim that may be made by its manufacturer, is not guaranteed or endorsed by the publisher.

## Abbreviations

GC, gastric cancer; DFS, disease-free survival; OS, overall survival; TNM, tumor–node–metastasis; CEA, carcinoembryonic antigen; CIs, confidence intervals; AIC, Akaike information criterion; HRs, hazard ratios; CSA, tumor cross-sectional area; NLR, neutrophil to lymphocyte ratio; PLR, platelet to lymphocyte ratio; VIMP, variable importance; ROC, receiver operating characteristic curve; AUC, area under curve.
