# Supplementary material for: Construction and Validation of Convenient Clinicopathologic Signatures for Predicting the Prognosis of Stage I-III Gastric Cancer
Source: Front Oncol. 2022 Mar 24;12:848783. doi: 10.3389/fonc.2022.848783 (PMC8987912; doi:10.3389/fonc.2022.848783)
Supplement: Supplementary file 1 [file DataSheet_1.docx]

Supplementary figure 1. Study design.





Supplementary figure 2. The VIMP values of all the variables are included in our signature. (a) The VIMP values for DFS. (b) The VIMP values for OS.


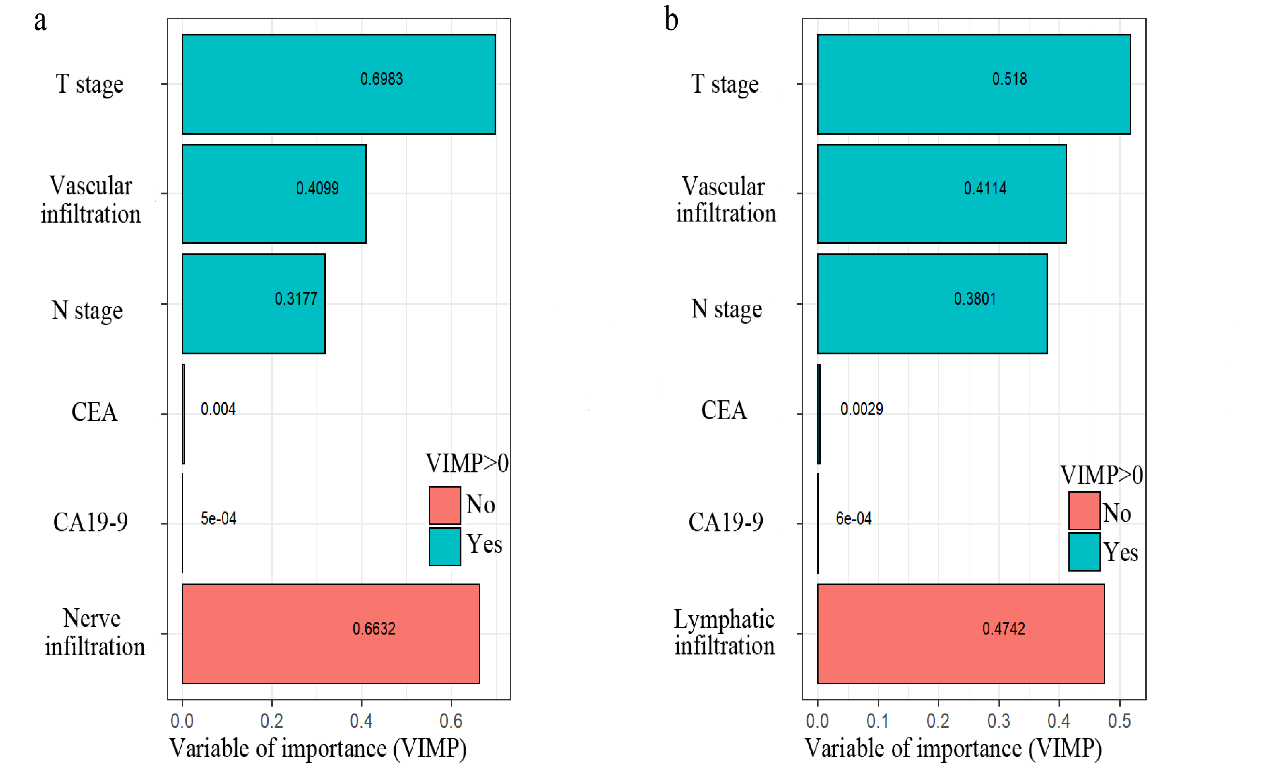


Supplementary figure 3. Kaplan-Meier curves for differences in patient survival in high-, intermediate-, and low-risk groups from external cohort validation. (a) Kaplan-Meier curves for DFS. (b) Kaplan-Meier curves for OS.


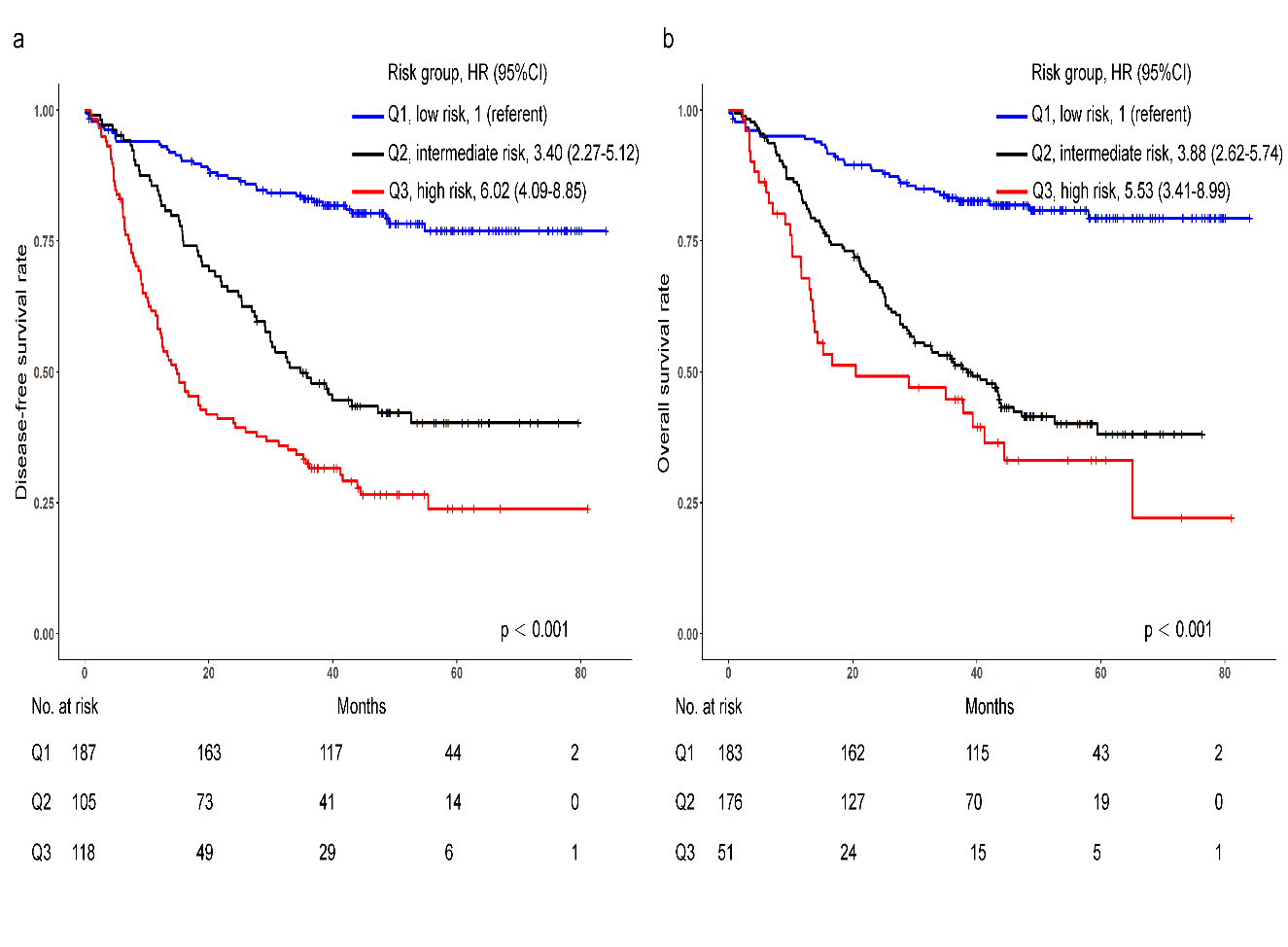


Supplementary Table 1 Patient demographics and clinicopathologic data of internal training cohort

| Variables | Patients  (N=585) |
| --- | --- |
| Gender |  |
| Male | 406 (69.4%) |
| Female | 179 (30.6%) |
| Median follow-up months | 45.0 |
| Median age (range) | 63 (21-93) |
| T stage |  |
| T1+T2 | 184 (31.4%) |
| T3 | 125 (21.4%) |
| T4 | 276 (47.2%) |
| N stage |  |
| N0 | 261 (44.6%) |
| N1 | 84 (14.4%) |
| N2 | 97 (16.6%) |
| N3 | 143 (24.4%) |
| Pathological stage |  |
| I | 154 (26.3%) |
| II | 165 (28.2%) |
| III | 266 (45.5%) |
| Tumor CSA (mean ± SD) | 12.00 ± 24.52 cm^2^ |
| Pathological type |  |
| Adenocarcinoma | 557 (95.2%) |
| Mucinous Adenocarcinoma and Signet-ring cell carcinoma | 28 (4.8%) |
| Degree of differentiation |  |
| Moderate and well | 103 (17.6%) |
| Poor | 482 (82.4%) |
| Lymphatic infiltration |  |
| Present | 386 (66.0%) |
| Absent | 199 (34.0%) |
| Vascular infiltration |  |
| Present | 141 (24.1%) |
| Absent | 444 (75.9%) |
| Nerve infiltration |  |
| Present | 461 (78.8%) |
| Absent | 124 (21.2%) |
| Median Ki67 (Range) | 55% (5%-95%) |
| NLR (mean ± SD) | 2.92 ± 2.85 |
| PLR (mean ± SD) | 158.12 ± 84.25 |
| CEA value (mean ± SD, ng/ml) | 8.83 ± 41.95 |
| CA125 value (mean ± SD, U/ml) | 17.47 ± 35.33 |
| CA19-9 value (mean ± SD, U/ml) | 66.69 ± 256.69 |
| Metastasis or recurrence |  |
| Yes | 203 (34.7%) |
| No | 382 (65.3%) |
| Survival status |  |
| Alive | 355 (60.7%) |
| Dead | 230 (39.3%) |

Supplementary table 2 Simulated Annealing Arithmetic to select variables to construct a model for DFS

| **Variables** | **Coef** | **S.E.** | **Wald** | **Pr (> \|Z\|)** |
| --- | --- | --- | --- | --- |
| T stage | 0.6983 | 0.1222 | 5.71 | < 0.0001 |
| N stage | 0.3177 | 0.0676 | 4.70 | < 0.0001 |
| Vascular infiltration | 0.4099 | 0.1563 | 2.62 | 0.0087 |
| Nerve infiltration | -0.6632 | 0.3629 | -1.83 | 0.0676 |
| CEA | 0.004 | 0.0010 | 4.06 | < 0.0001 |
| CA19-9 | 0.0005 | 0.0002 | 3.12 | 0.0018 |
| C-index | 0.7502 | | | |

Supplementary table 3 Simulated Annealing Arithmetic to select variables to construct a model for OS

| **Variables** | **Coef** | **S.E.** | **Wald** | **Pr (> \|Z\|)** |
| --- | --- | --- | --- | --- |
| T stage | 0.5180 | 0.0896 | 5.78 | < 0.0001 |
| N stage | 0.3801 | 0.0737 | 5.16 | < 0.0001 |
| Lymphatic infiltration | -0.4742 | 0.2168 | -2.19 | 0.0287 |
| Vascular infiltration | 0.4114 | 0.1483 | 2.77 | 0.0055 |
| CEA | 0.0029 | 0.0009 | 3.05 | 0.0023 |
| CA19-9 | 0.0006 | 0.0002 | 3.42 | 0.0006 |
| C-index | 0.7341 | | | |

Supplementary Table 4 Univariate Cox regression analyses to validate our signatures (external validation cohort, NA, not available)

| **Variables** | **DFS, p-value** | **OS, p-value** |
| --- | --- | --- |
| T stage | 0.000 | < 0.001 |
| N stage | 0.000 | < 0.001 |
| Lymphatic infiltration | NA | 0.000 |
| Vascular infiltration | 0.017 | 0.021 |
| Nerve infiltration | 0.018 | NA |
| CEA | 0.000 | 0.001 |
| CA19-9 | 0.248 | 0.278 |
